# Mental Health Literacy, Anxiety, and Insomnia in Chinese Chronically Ill Older Adult‐Caregiver Dyads: Actor‐Partner Interdependence Moderation Model

**DOI:** 10.1111/famp.70077

**Published:** 2025-10-26

**Authors:** Xinyu Fan, Huiqiong Zheng, Shibin Wang, Wenyan Tan, Jing Liao

**Affiliations:** ^1^ Department of Medical Statistics, School of Public Health Sun Yat‐Sen University Guangzhou China; ^2^ Sun Yat‐Sen Global Health Institute, Institute of State Governance Sun Yat‐Sen University Guangzhou China; ^3^ Guangdong Mental Health Center, Guangdong Provincial People's Hospital (Guangdong Academy of Medical Sciences) Southern Medical University Guangzhou China

**Keywords:** actor‐partner interdependence moderation model, anxiety, caregivers, insomnia, mental health literacy, older adults

## Abstract

Anxiety and insomnia are correlated in older adults and their caregivers, yet the moderating role of mental health literacy (MHL) is unclear. This study aimed to explore dyadic effects of anxiety on insomnia among Chinese chronically ill older adults and family caregivers across age groups and whether MHL moderates these effects. Data came from 1033 dyads of older adults and their family caregivers in China through the Guangdong Mental Health Survey. Anxiety was assessed with the Generalized Anxiety Disorder‐7, insomnia with the Insomnia Severity Index, and MHL with the Chinese National Mental Health Literacy Scale (consisting of mental health knowledge, attitudes, and capacity). The Actor‐Partner Interdependence Moderation Model was applied for analysis. Young caregivers' mental health attitudes, *β* = −0.558, *p* = 0.002, mitigated the effect of their anxiety on their insomnia, while older adults' mental health knowledge, *β* = 0.428, *p* = 0.019, enhanced this relationship. Older adults' mental health attitudes, *β* = −0.731, *p* = 0.004, reduced the impact of middle‐aged caregivers' anxiety on the latter's insomnia. Middle‐aged caregivers' mental health capacity, *β* = −0.367, *p* = 0.004, attenuated the effect of older adults' anxiety on caregivers' insomnia. No significant moderating effects were observed in the dyad group of older adults and older caregivers. Within caregiving dyads, enhancing MHL can potentially reduce the impact of anxiety on insomnia. Interventions aimed at improving the mental health attitudes of older adults and caregivers are more likely to alleviate anxiety and insomnia than mental health knowledge and capacity.

## 1 Introduction

China has an aging society, and the burden of disease among older adults has increased (Hu et al. 2021). Anxiety disorders are common mental disorders, both among older and younger adults. A previous study reported a 12‐month prevalence of 4.7% among Chinese adults aged over 65, and 4.3% to 4.8% among those aged 18–49 (Huang et al. 2019). Although the rate of anxiety disorders appears low, it ranks first among all mental disorders. Further, when considering China's population of 1.4 billion, the prevalence value indicates that there are a very large number of affected individuals. Sleep disorders are another common mental health problem. A meta‐analysis showed that 35.9% of Chinese older adults suffer from sleep disorders (Lu et al. 2019), a large proportion of adults as well as a large raw number of individuals given China's population size.

These mental health problems bring about substantial disease burden and are more prominent among older adults with chronic disease. A higher prevalence of anxiety symptoms (85%) and sleep disorders (44%) is observed among older adults suffering from chronic diseases in comparison to those of a healthier constitution, with corresponding figures of 23% and 15%, respectively (Xie et al. 2023; Yan et al. 2022). In China, family members primarily look after older adults (Chen et al. 2020). Mental health problems not only reduce quality of life for older adults but also increase caregiver stress in family members. Research shows that anxiety and sleep problems affect both older adults with chronic diseases and their family caregivers (Bouchard et al. 2021; Ivziku et al. 2019).

The positive association between anxiety and insomnia has been well established in both older adults and caregivers, but the association is limited at an individual level. Prior studies have shown that anxiety is associated with insomnia in older adults (Dragioti et al. 2017; Magee and Carmin 2010) and in caregivers (Starr et al. 2023), but few have examined the interdependence within caregiver‐care recipient dyads. In caregiving relationships, anxiety experienced by either the older adult or the caregiver may influence the well‐being of both. The theory of Dyadic Illness Management proposes that dyads, such as spouses or parent‐children, experience illness as an interdependent team and that the health of the dyad covaries (Lyons and Lee 2018). Analysis of dyadic data indicates an interpersonal correlation of emotion between older adults and caregivers (Ivziku et al. 2019). Building on this framework, Ding et al. (2023) applied the actor‐partner interdependence model (APIM) to explore the dyadic effects of anxiety on insomnia in Chinese older adults and their caregivers. Their findings showed that older adults' anxiety had an influence on caregivers' insomnia, highlighting the presence of dyadic effect in these relationships.

The dyadic effects of anxiety on insomnia in dyad groups of older adults and caregivers from different age groups may vary. However, Ding et al. (2023) did not consider that caregivers of different ages may experience different effects. Epidemiological characteristics of insomnia suggest that age is a key factor influencing insomnia (Roth and Roehrs 2003). Caregiver role further contributes to these differences. Spouses who are older caregivers are more likely to experience physical strain and caregiver burden (Koumoutzis et al. 2021), while adult children who are also younger caregivers often face depression and loneliness (Musich et al. 2017). One study suggests that spousal caregivers have some specific experiences (changing identity of feeling and being married; may become alone) compared to other family caregivers (Hennings and Froggatt 2019). Thus, caregivers of different ages face distinct challenges and stress levels. Therefore, in caring for older adults with chronic diseases, we cannot ignore the influence of age or the role of the caregivers (spouses or adult children) in these dyadic effects of anxiety on insomnia.

Mental health literacy (MHL), which is closely linked to the occurrence of psychological problems, is a concept generally divided by Kutcher et al. (2016) into three dimensions: mental health knowledge (MHK), mental health attitudes (MHA), and mental health capacity (MHC). MHK encompasses basic knowledge and principles of mental health, mental illnesses, and their treatment, crisis intervention, and suicide prevention (Bjørnsen et al. 2017; Jorm et al. 1997). MHA involves attitudes towards the prevention and treatment of mental illnesses, reduction of stigma, and attitudes towards psychological help‐seeking (Jorm et al. 1997; Jorm 2012). Some individuals hold negative attitudes, perceiving people with mental illness as dangerous, shameful, or beyond recovery—beliefs that contribute to social rejection and may hinder help‐seeking (Angermeyer et al. 2009; Mojtabai 2010). In contrast, others hold positive or neutral attitudes, recognizing mental illness as a health problem requiring timely treatment or responding without undue fear or aversion. MHC refers to the ability to access mental health information, recognize symptoms, offer psychological first aid, and regulate emotions (Jorm 2012; O'Connor et al. 2014). MHL has been increasingly recognized for improving the health outcomes of populations (Kelly et al. 2007). Regarding the MHL related to various anxiety disorders, age is an influential factor, with younger people having better MHL compared to older ones (Hadjimina and Furnham 2017). While high MHL is associated with lower levels of anxiety, insomnia, and other psychological symptoms, the specific mechanisms by which its dimensions exert protective effects remain unclear (Jorm 2012; Kelly et al. 2007). A study has shown the moderator role of MHL in the relationship between bullying victimization and anxiety, and it can mitigate the positive relationship (Huang et al. 2023). Although numerous studies support the positive relationship between anxiety and insomnia, whether MHL moderates this association has not been fully explored—particularly in dyads of older adults and their caregivers.

The study aimed to explore the dyadic effects of anxiety on insomnia among Chinese older adults with chronic diseases and their family caregivers of different age groups, as well as the moderating role of MHL (mental health knowledge, attitudes, and capacity) in these dyadic effects within different dyad groups of older adults and caregivers.

## 2 Method

### 2.1 Ethics

Ethics approval was obtained from the Ethics Committee of the Guangdong Provincial People's Hospital, Guangdong Academy of Medical Sciences (Reference number: KY2020‐268‐01). All procedures were conducted in accordance with the Declaration of Helsinki. All participants in this study provided informed consent.

### 2.2 Study Design and Participants

To assess MHL and the prevalence of conditions such as depression and anxiety disorders among adults in Guangdong Province, the Guangdong Mental Health Survey was conducted across all 21 prefecture‐level cities. It adopted the multistage stratified cluster random sampling method to select community‐dwelling residents between September and December 2021. In the first stage, 3–5 districts or counties were selected from each prefecture‐level city using the probability proportional to size (PPS) method. In the second stage, 1–4 subdistricts or towns were sampled from each selected district or county, again based on population size. Subsequently, 2–4 neighborhood/village committees were selected from each subdistrict/town using the PPS method. Within each committee, 50 households were chosen through systematic sampling. Finally, one adult (aged 18 or older) was randomly selected from each household to complete the survey. All selected households were informed of the survey's purpose (to understand the mental health status of adult residents across the province and analyze psychosocial service needs for better targeted mental health services) and given the option to decline participation. For those who consented, personal information was anonymized to protect privacy, and participants were assured they could withdraw from the survey at any time.

If a resident aged 65 or older was selected, that individual's primary adult family caregiver was also invited to participate in the survey. Caregivers were informed of the survey's purpose and were free to decline or withdraw at any time. If the older adult was residing in a nursing home or hospitalized during the survey period, another resident was re‐selected as a substitute. Face‐to‐face interviews covering demographic, health‐related, and mental health information were conducted by trained interviewers using electronic structured questionnaires at local health service centers. A total of 4018 older adults (aged 65 and above) and their primary family caregivers were included in this study. Primary family caregivers were defined as family members who provide the majority of daily care for the older adult.

Participants were older adult‐caregiver dyads drawn from the above survey in this study. Eligible older adults were defined as: (a) community‐living resident aged ≥ 65 years; (b) have at least one chronic disease (such as diabetes, hypertension, COPD, etc.); (c) not in a caregiver role; (d) their primary caregivers completed questionnaires data. A total of 1534 primary caregivers of older adults completed the questionnaires, out of a total of 4018 older adults, with a 38.2% response rate. Of these 1534 older adults, those being in a caregiver role were excluded (*n* = 17), and then those without chronic disease were excluded (*n* = 484). Finally, 1033 pairs of older adults and their primary caregivers were included in this study for dyadic analysis.

### 2.3 Measurements

The anxiety symptoms of older adults and their caregivers in the past 2 weeks were evaluated using the Generalized Anxiety Disorder‐7 (GAD‐7; Spitzer et al. 2006). The GAD‐7 consists of 7 items. Respondents reported their agreement with each item on a scale from 0 (*not at all*) to 3 (*nearly every day*). The total score, ranging from 0 to 21, was calculated by summing the responses to each item. The sample in this study showed high internal consistency, with *α* = 0.92 for the older adults and 0.89 for the caregivers.

The participants reported their insomnia symptoms in the past 2 weeks using the Insomnia Severity Index (ISI; Bastien et al. 2001). This scale consists of 7 items, each rated on a 5‐point Likert scale from 0 to 4. Items 1–3: 0 = *no problem*, 4 = *very severe problem*; item 4: 0 = *very satisfied*, 4 = *very dissatisfied*; items 5–7: 0 = *not at all*, 4 = *very much*. The total score ranges from 0 to 28, with higher scores indicating greater insomnia severity. Internal consistency coefficients were high for both older adults (*α* = 0.93) and caregivers (*α* = 0.89).

MHL was assessed using the Chinese National Mental Healthy Literacy Scale (see Appendix S1), the official instrument designated by the National Health Commission of the People's Republic of China for monitoring indicators in “Mental Health Promotion Action.” This scale is used to determine whether individuals meet the national standard for MHL. The scale comprises three sections: judgment questions, self‐assessment questions, and case questions, which respectively measure MHK, MHA, and MHC. The first section includes 20 true‐or‐false items covering basic mental health concepts (e.g., “anxiety and other emotions are harmful”, “the earlier the treatment of mental illness, the better”, “bad mood may cause physical disease”). Scores range from 0 to 100, with ≥ 80 indicating that the MHK meets the standard. The second section includes 8 self‐report items evaluating participants' attitudes and confidence related to mental health (e.g., “I know how to acquire mental health knowledge”), rated on a four‐point Likert scale from *never* to *always*. Scores range from 8 to 32, with ≥ 24 indicating that the MHA meets the standard. The third section presents two case vignettes (each with 4 items) describing individuals experiencing psychological distress (e.g., a college student with persistent sadness, insomnia, and loss of appetite). Participants were asked to identify the likely mental illness and appropriate responses. This section has a total score of 0 to 40, with ≥ 28 showing that the MHC meets the standard.

Additionally, we measured the demographic variables, including age, sex, education level, region (urban/rural), monthly income, alcohol drinking habits, tea drinking habits, and the number of chronic diseases by using a self‐administered questionnaire.

### 2.4 Statistical Analysis

**FIGURE 1:**
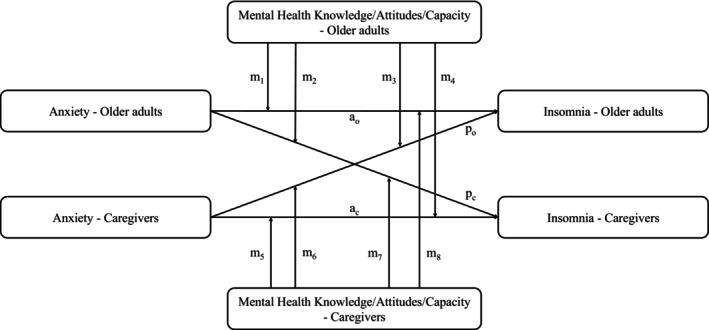
Framework of the actor–partner interdependence moderation model. a, actor effect; C, Caregivers; m, moderator effect; O, Older adults; p, partner effect.

Caregivers were categorized into three age groups: young (18–44 years), middle‐aged (45–64 years), and older caregivers (≥ 65 years). Descriptive statistics including mean, standard deviation, frequency, and percentage were used to summarize the demographic characteristics and main variables of older adults and caregivers. Paired sample *t*‐tests, Chi‐square test, or Wilcoxon paired test was used to measure the differences between older adults and overall caregivers for all variables. One‐way ANOVA, Chi‐square test, or Kruskal‐Wallis test was used to examine the differences in all variables across caregiver age groups. To present the actor and partner effects on insomnia, APIM (Cook and Kenny 2005) was used. Actor effects represent the relationship between an individual's anxiety and their own insomnia, and partner effects represent the relationship between an individual's anxiety and the other dyad member's insomnia. Model 1 was a crude model without any covariates, and Model 2 was based on Model 1 adjusted for age, sex, region, and the number of chronic diseases. Subsequently, Model 3, building upon the adjustments made in Model 2, further adjusted for MHK, MHA, and MHC. These were analyzed separately in different dyad groups of older adults and caregivers. We finally adopted the actor‐partner interdependence moderation model (APIMoM; Garcia et al. 2015) by using a two‐intercept approach to investigate the moderator role of MHK, MHA, and MHC (as observed in the conceptual model from Figure 1). The independent variable of anxiety was centralized. Given the original purpose of the MHL scale—to assess whether individuals meet the national standards—and considering potential policy implications, MHK, MHA, and MHC were treated as binary moderator variables (*qualified* vs. *unqualified*) to enhance interpretability and applicability of findings. Analyses were conducted in R version 4.4.0 (R Core Team, Vienna, Austria), and all the tests were two‐sided, with statistical significance set at 0.05.

## 3 Results

### 3.1 Characteristics of Older Adults and Caregivers

A total of 1033 older adults and their primary family caregivers participated in the study, yielding a sample of 2066 individuals. As shown in Table 1, the mean age of older adults was 73.21 (SD = 6.44), and 55.5% were female. Caregivers were categorized by age into young (18–44 years, *n* = 565), middle‐aged (45–64 years, *n* = 367), and older (≥ 65 years, *n* = 101) groups, with mean ages of 37.02 (SD = 5.72), 52.61 (SD = 5.95), and 71.23 (SD = 4.93), respectively. The majority of caregivers were also female, comprising 56.3% of young, 59.7% of middle‐aged, and 63.4% of older caregiver groups. Significant differences were found between older adults and caregivers in both raw scores and qualification rates for MHK, MHA, and MHC, except for MHK qualification rate (*p* = 0.144). Among caregiver age groups, only MHK scores differed significantly (*p* < 0.001), with young caregivers scoring highest (63.14) and older caregivers lowest (56.29). Regarding anxiety, 90.0% of older adults reported normal levels, while 8.2% had mild anxiety, 1.0% moderate, and 0.8% severe anxiety. No significant differences in anxiety levels were observed between older adults and caregivers overall, but differences were significant across caregiver age groups. For insomnia, 80.9% of older adults reported no symptoms, 14.9% mild, 3.7% moderate, and 0.5% severe symptoms. Additional sample characteristics are presented in Table 1.

**TABLE 1:** Sample characteristics.

Characteristics	Older adults^a^ (*N* = 1033)	Family caregivers (*N* = 1033)			*p*_1_ ^b^	*p*_2_ ^c^
		Young caregivers^a^ (*n* = 565)	Middle‐aged caregivers^a^ (*n* = 367)	Older caregivers^a^ (*n* = 101)		
Age	73.21 (6.44)	37.02 (5.72)	52.61 (5.95)	71.23 (4.93)	**< 0.001**	**< 0.001**
Sex						
Male	460 (44.5%)	247 (43.7%)	148 (40.3%)	37 (36.6%)	0.214	0.318
Female	573 (55.5%)	318 (56.3%)	219 (59.7%)	64 (63.4%)		
Region						
Urban	554 (53.6%)	315 (55.8%)	178 (48.5%)	64 (63.4%)	0.895	**0.013**
Rural	479 (46.4%)	250 (44.2%)	189 (51.5%)	37 (36.6%)		
Education level						
Primary school and below	632 (61.2%)	14 (2.5%)	71 (19.3%)	57 (56.4%)	**< 0.001**	**< 0.001**
Junior high school	224 (21.7%)	129 (22.8%)	140 (38.1%)	26 (25.7%)		
Senior high school	135 (13.1%)	145 (25.7%)	86 (23.4%)	13 (12.9%)		
College and above	42 (4.1%)	277 (49.0%)	70 (19.1%)	5 (5.0%)		
Income (RMB)						
< 3500	632 (61.2%)	208 (36.8%)	198 (54.0%)	69 (68.3%)	**< 0.001**	**< 0.001**
3500–5999	244 (23.6%)	180 (31.9%)	115 (31.3%)	20 (19.8%)		
6000–9000	77 (7.5%)	78 (13.8%)	25 (6.8%)	9 (8.9%)		
> 9000	80 (7.7%)	99 (17.5%)	29 (7.9%)	3 (3.0%)		
Alcohol‐drinking habits						
No	964 (93.3%)	508 (89.9%)	337 (91.8%)	95 (94.1%)	**0.050**	0.320
Yes	69 (6.7%)	57 (10.1%)	30 (8.2%)	6 (5.9%)		
Tea‐drinking habits						
No	570 (55.2%)	274 (48.5%)	159 (43.3%)	62 (61.4%)	**0.001**	**0.005**
Yes	463 (44.8%)	291 (51.5%)	208 (56.7%)	39 (38.6%)		
Number of chronic diseases						
0	0 (0%)	441 (78.1%)	206 (56.1%)	35 (34.7%)	**< 0.001**	**< 0.001**
1	534 (51.7%)	99 (17.5%)	91 (24.8%)	33 (32.7%)		
≥ 2	499 (48.3%)	25 (4.4%)	70 (19.1%)	33 (32.7%)		
Mental health knowledge	56.63 (17.04)	63.14 (15.11)	61.68 (15.04)	56.29 (14.88)	**< 0.001**	**< 0.001**
Unqualified	927 (89.7%)	487 (86.2%)	325 (88.6%)	94 (93.1%)	0.144	0.126
Qualified	106 (10.3%)	78 (13.8%)	42 (11.4%)	7 (6.9%)		
Mental health attitudes	28.16 (3.37)	28.81 (3.03)	29.26 (2.96)	29.08 (3.62)	**< 0.001**	0.088
Unqualified	99 (9.6%)	31 (5.5%)	18 (4.9%)	5 (5.0%)	**< 0.001**	0.919
Qualified	934 (90.4%)	534 (94.5%)	349 (95.1%)	96 (95.0%)		
Mental health capacity	29.21 (7.23)	31.10 (6.68)	30.73 (6.81)	29.76 (6.62)	**< 0.001**	0.168
Unqualified	405 (39.2%)	168 (29.7%)	122 (33.2%)	35 (34.7%)	**< 0.001**	0.407
Qualified	628 (60.8%)	397 (70.3%)	245 (66.8%)	66 (65.3%)		
Anxiety	0 (0, 1)	0 (0, 2)	0 (0, 1)	0 (0, 0)	0.871	**0.031**
Normal (0–4)	930 (90.0%)	511 (90.4%)	324 (88.3%)	100 (99.0%)	0.387	**0.003**
Mild anxiety (5–9)	85 (8.2%)	48 (8.5%)	29 (7.9%)	1 (1.0%)		
Moderate anxiety (10–14)	10 (1.0%)	5 (0.9%)	11 (3.0%)	0 (0%)		
Severe anxiety (15–21)	8 (0.8%)	1 (0.2%)	3 (0.8%)	0 (0%)		
Insomnia	2 (0, 6)	1 (0, 4)	1 (0, 5)	2 (0, 5)	**< 0.001**	0.373
Normal (0–7)	836 (80.9%)	510 (90.3%)	310 (84.5%)	89 (88.1%)	**< 0.001**	**0.001**
Mild insomnia (8–14)	154 (14.9%)	54 (9.6%)	46 (12.5%)	10 (9.9%)		
Moderate insomnia (15–21)	38 (3.7%)	1 (0.2%)	11 (3.0%)	2 (2.0%)		
Severe insomnia (22–28)	5 (0.5%)	0 (0%)	0 (0%)	0 (0%)		

*Note:* Findings significant at the *p* ≤ 0.05 level are shown in bold.

^a^ Continuous variable was presented as *M* (SD) except for anxiety and insomnia; Anxiety and insomnia were presented as *Md* (IQR); Categorical variable was presented as *n* (*%*).

^b^ Significance of differences between older adults and overall family caregivers by using paired sample *t*‐test, chi‐square test, or Wilcoxon paired test.

^c^ Significance of differences between young, middle‐aged, and older caregivers by using one‐way ANOVA, chi‐square test, or Kruskal‐Wallis test.

### 3.2 Actor‐Partner Interdependence Model

Table 2 presents the results of APIM. In Model 1 (unadjusted), anxiety was positively associated with individuals' own insomnia across all three dyad groups, indicating significant actor effects: higher anxiety levels were linked to greater insomnia within both older adults and caregivers. A significant partner effect was observed only in the dyad group of older adults and young caregivers, where older adults' anxiety was positively related to young caregivers' insomnia, *β =* 0.099, *p =* 0.048. Model 2 adjusted for covariates including age, sex, region, and number of chronic diseases. Actor effects remained significant and positive across all dyad groups, but no significant partner effects were detected. Model 3 further adjusted for MHK, MHA, and MHC. The results remained consistent with Model 2: actor effects persisted across all groups, while no partner effects were observed. Full model results are reported in Table 2.

**TABLE 2:** Actor effect and partner effect of older adults and family caregivers of different ages for anxiety and insomnia.

Effect	Model 1^a^			Model 2^b^			Model 3^c^		
	*β*	SE	*p*	*β*	SE	*p*	*β*	SE	*p*
Young caregiver (18–44 years old, *n* = 565)									
Actor effect									
Older adult	**0.859**	**0.067**	**< 0.001**	**0.782**	**0.069**	**< 0.001**	**0.739**	**0.069**	**< 0.001**
Caregiver	**0.646**	**0.049**	**< 0.001**	**0.618**	**0.049**	**< 0.001**	**0.600**	**0.050**	**< 0.001**
Partner effect									
Older adult	−0.002	0.068	0.979	−0.007	0.067	0.918	−0.026	0.067	0.696
Caregiver	**0.099**	**0.048**	0.**041**	0.076	0.048	0.116	0.072	0.049	0.141
Middle‐aged caregiver (45–64 years old, *n* = 367)									
Actor effect									
Older adult	**0.862**	**0.076**	**< 0.001**	**0.795**	**0.075**	**< 0.001**	**0.782**	**0.077**	**< 0.001**
Caregiver	**0.815**	**0.065**	**< 0.001**	**0.745**	**0.066**	**< 0.001**	**0.707**	**0.067**	**< 0.001**
Partner effect									
Older adult	−0.081	0.081	0.317	−0.061	0.079	0.442	−0.069	0.081	0.391
Caregiver	0.068	0.061	0.263	0.057	0.059	0.340	0.057	0.060	0.344
Older caregiver (over 65 years old, *n* = 101)									
Actor effect									
Older adult	**1.075**	**0.165**	**< 0.001**	**1.055**	**0.167**	**< 0.001**	**1.076**	**0.175**	**< 0.001**
Caregiver	**1.000**	**0.277**	**< 0.001**	**1.059**	**0.275**	**< 0.001**	**1.080**	**0.291**	**< 0.001**
Partner effect									
Older adult	−0.209	0.266	0.432	−0.133	0.267	0.620	−0.126	0.292	0.665
Caregiver	−0.088	0.172	0.609	−0.090	0.167	0.592	−0.008	0.170	0.964

*Note:* Findings significant at the *p* ≤ 0.05 level are shown in bold.

^a^ Model 1 was a crude model to examine the relationship between anxiety and insomnia without covariates.

^b^ Model 2 was a covariate‐adjusted model based on model 1 after controlling for age, sex, region, and the number of chronic diseases.

^c^ Model 3 incorporated mental health knowledge, attitudes, and capacity as covariates based on model 2.

### 3.3 Actor‐Partner Interdependence Moderation Model

**FIGURE 2:**
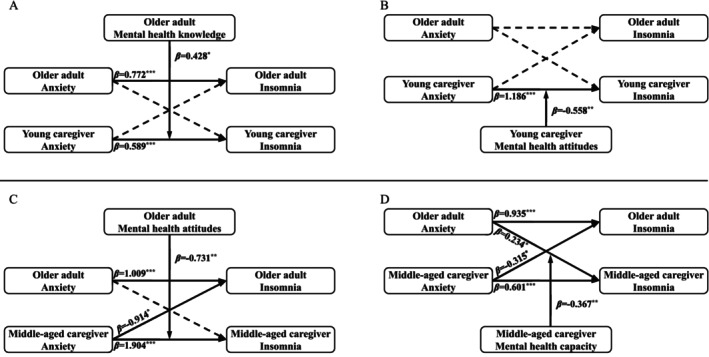
Actor–partner interdependence moderation model of anxiety and insomnia within different older adult–caregiver dyads. Standardized coefficients (*β*) are presented on straight lines with single arrows. A solid line and dotted line represent significant paths and non‐significant ones respectively. *p < 0.05. **p < 0.01. ***p < 0.001.

The significant moderating effect of MHK, MHA, MHC, and the impact of the dyadic interaction between older adults and caregivers were depicted in Figure 2. The detailed results of APIMoM were shown in Table B1 (see Appendix S2).

In the dyad group comprising older adults and young caregivers, both MHK and MHA demonstrated significant moderating effect. Young caregivers' anxiety, *β =* 0.589, *p* < 0.001, was positively linked to their own insomnia, and this effect was strengthened when the older adults had qualified MHK, *β =* 0.428, *p* = 0.019. Young caregivers' MHA, *β =* −0.558, *p* = 0.002, could mitigate their own actor effect, *β =* 1.186, *p* < 0.001, meaning young caregivers with qualified MHA had a weaker positive effect of anxiety on insomnia compared to those with unqualified MHA.

In the dyad group of older adults and middle‐aged caregivers, older adults' MHA, *β* = −0.731, *p* = 0.004, moderated the caregivers' actor effect, *β =* 1.904, *p* < 0.001, attenuating the impact of caregivers' anxiety on their own insomnia. Additionally, a significant older adults' partner effect was observed in this model, *β =* −0.914, *p =* 0.046, indicating that caregivers' anxiety was negatively associated with insomnia in older adults. Furthermore, middle‐aged caregivers' MHC, *β* = −0.367, *p* = 0.004, moderated the impact of older adults' anxiety on caregivers' insomnia (partner effect: *β* = 0.234, *p* = 0.023), suggesting that caregivers with qualified MHC could reduce the impact of that partner effect. In this model including MHC, both partner effects were significant but in opposite directions: older adults' anxiety increased caregivers' insomnia, *β* = 0.234, *p* = 0.023, while caregivers' anxiety was associated with decreased insomnia in older adults, *β* = −0.315, *p* = 0.041.

In the dyad group of older adults and older caregivers, we did not find any moderating effects of MHK, MHA, and MHC.

## 4 Discussion

In the results of APIM, we found that anxiety in older adults and caregivers of different ages had an influence on their own insomnia (actor effect); older adults' anxiety had an influence on young caregivers' insomnia (partner effect). In the APIMoM, we saw that MHA played a moderating role in the effect of anxiety on insomnia in the dyad groups of older adults and young/middle‐aged caregivers. The distinction lies in whose MHA moderates either the actor or partner effect within the dyad group. MHK moderated the effect of anxiety on insomnia only in the dyad group of older adults and young caregivers, whereas MHC moderated that effect only in the dyad group of older adults and middle‐aged caregivers.

The significant actor effects observed in the APIM—where both older adults' and caregivers' anxiety were associated with their own insomnia—are consistent with previous findings (Chan et al. 2017; Ding et al. 2023). However, partner effects emerged only in the dyad group of older adults and young caregivers. The limited presence of partner effects may be explained by the fact that insomnia in both caregivers of different ages and older adults was mainly due to their own anxiety during the COVID‐19 pandemic. Older adults suffer from chronic diseases and are more likely not to sense the emotions of their caregivers but rather the care activities of caregivers. Caregivers were more anxious about financial strain rather than the emotions of older adults during the COVID‐19 pandemic (Freisthler et al. 2021). Interestingly, in the APIMoM that included MHC, partner effects were observed in opposite directions within the middle‐aged caregiver dyads: older adults' anxiety was positively associated with caregivers' insomnia, while caregivers' anxiety was negatively associated with insomnia in older adults. One possible explanation is that older adults experiencing heightened anxiety about illness or recovery may require more care, especially at night, thereby disrupting caregivers' sleep. Conversely, while caregivers may experience anxiety due to prolonged caregiving demands, their efforts to ensure adequate care may reassure older adults and improve their sleep quality. Further research needs to combine qualitative research to confirm our speculation.

MHA was found to have a moderating effect in the dyad groups of older adults and young/middle‐aged caregivers. Young caregivers' MHA could moderate the positive effect of their anxiety on their own insomnia. There are many sources of anxiety for young caregivers, including financial concerns, caring for their own children and parents, and work‐related pressures (Majeed et al. 2018). The mitigation of anxiety effects can be achieved by improving their own MHA. The influence of middle‐aged caregivers' anxiety on their personal insomnia could be mitigated by older adults' MHA. Middle‐aged caregivers, being more mature than young caregivers and having adult children, may find that caring for their senior family members becomes a major responsibility and may value some views of their seniors. In addition, middle‐aged caregivers have more contact with older adults and are more likely to use strategies of engagement and acceptance in their relationships with their parents (Wang et al. 2020), so older adults' MHA may play a moderating role.

MHK and MHC were found to have a less significant moderating effect than MHA in different dyad groups of older adults and caregivers. One potential explanation for this is that the overall MHK and MHC levels of older adults and caregivers were low in this study. Unlike MHA, we found that older adults with qualified MHK showed a stronger positive association between young caregivers' anxiety and their own insomnia, compared to those with unqualified MHK. This may be because older adults with qualified MHK know the basics of mental health, understand the possible dangers of anxiety, and subsequently educate the young caregivers. However, young caregivers face additional pressures and are less likely to follow the advice of older adults, which can exacerbate their anxiety (Zhang and Wiebe 2022). On the other hand, there may be differences in the perceptions of older adults and young caregivers, which can easily lead to conflicts in the interaction (Subramaniam and Mehta 2024), and this, in turn, can increase the anxiety of young caregivers. Middle‐aged caregivers' MHC played a moderating role in middle‐aged caregivers' partner effect. MHC includes skills such as access to mental health information, identification of specific mental illnesses, psychological first aid, and emotion regulation (Jorm 2012). In other words, it is a response to seeking mental health help. The moderating effect of MHC was only found in middle‐aged caregivers, which may be due to their having more leisure time than young caregivers, and their superior ability to use it more effectively than older caregivers (Anderson et al. 2013).

In our sample, both older adults and caregivers reported relatively low levels of anxiety and insomnia. One explanation may be that participants were general community residents and did not include closed institutional communities (e.g., universities or military settings) and hospitalized patients. Additionally, the GAD‐7 and ISI scales assessed symptoms over the preceding 2 weeks, during which participants' chronic health conditions and emotional states may be relatively stable. Social stigma surrounding mental health may also have contributed to underreporting. The limited symptom severity observed suggests that most participants fell within the mild or subclinical range. This restricted variability may have attenuated our ability to detect strong associations between anxiety and insomnia, particularly if such associations are more evident at higher symptom levels. Consequently, while our findings offer insight into the early interplay between anxiety, insomnia, and MHL, they may not generalize to individuals experiencing clinically significant distress. Nevertheless, detecting significant associations even in a low‐symptom sample underscores the value of early psychological intervention—prior to the escalation of symptoms to clinical levels. Future research could incorporate wearable devices to enhance the objectivity of anxiety and insomnia assessments, and explore whether similar patterns emerge in populations with greater symptom severity.

We examined the moderating role of MHL across different dyad groups of older adults and caregivers, focusing on the distinct contributions of each member's MHL. Although evidence‐based interventions exist for caregivers, most are not tailored to the specific characteristics of the caregiver‐care recipient relationship. By exploring the unique roles and dynamics within caregiving dyads, this study provides a foundation for more targeted mental health interventions. Future research is needed to further elucidate the moderating mechanisms of MHL, in order to inform the development of interventions that are appropriately adapted to the needs of different dyad configurations.

This study has several limitations. First, the concept and measurement of MHL remain evolving and lack consensus. Although the connotations and dimensions of MHL are constantly being updated and expanded, there is no universally accepted framework, resulting in the proliferation of diverse instruments with inconsistent content and varying levels of reliability and validity (Jorm et al. 1997; O'Connor et al. 2014). Future research should work towards clarifying the conceptual structure of MHL and developing psychometrically robust tools to support more reliable assessments. Second, we categorized caregivers by age groups rather than by relationship role (e.g., spouse, adult children), which differs from the common approach in prior research (Pinquart and Sörensen 2011). This approach may obscure important role‐based differences in caregiving experiences and limits interpretation of how caregiver‐care recipient dynamics vary by relational context. Third, this study focused only on MHL and did not consider physical health literacy, but the two are mutually influential. Furthermore, although we adjusted for the number of chronic diseases in older adults, we did not consider the specific type of disease. Caregiver burden may differ depending on the condition being managed—for example, caregiving for individuals with schizophrenia may be more demanding than for those with chronic physical illnesses such as diabetes (Ampalam et al. 2012). Future studies could target specific disease groups and employ validated instruments to quantify caregiver burden more precisely. Finally, the findings are based on provincial data and may not be generalizable to other regions of China.

## 5 Conclusion

The findings highlight that improving MHL may mitigate the effects of anxiety on insomnia. Enhancing mental health attitudes, rather than solely focusing on knowledge and capacity, may be more effective in reducing the impact of anxiety on insomnia among caregivers and older adults with chronic diseases. Specifically, in dyad groups comprising young caregivers and older adults, enhancing the caregiver's mental health attitudes showed a moderating effect, while the same improvement in older adults did not yield the same result. In dyad groups with middle‐aged caregivers and older adults, enhancing older adults' mental health attitudes had a moderating effect. Tailored interventions are essential to address the caregiver–older adult relationship in managing insomnia among family caregivers, with a focus on different age groups and incorporating mood management strategies. For example, developing educational materials and support programs tailored to the specific needs and preferences of different age groups, considering factors such as communication styles, learning preferences, and generational differences.

## Conflicts of Interest

The authors declare no conflicts of interest.

## Supporting information


**Appendixes S1–S2.** Supporting Information.

## Data Availability

The data that support the findings of this study are available from the corresponding author upon reasonable request.
