# Supplementary material for: Mental Health Literacy, Anxiety, and Insomnia in Chinese Chronically Ill Older Adult‐Caregiver Dyads: Actor‐Partner Interdependence Moderation Model
Source: Fam Process. 2025 Oct 26;64(4):e70077. doi: 10.1111/famp.70077 (PMC12554632; doi:10.1111/famp.70077)
Supplement: Supplementary file 1 — Appendixes S1–S2. Supporting Information. [file FAMP-64-0-s001.docx]

**Appendix S1**

**Chinese National Mental Health Literacy Scale**

**Part 1: Judgement Questions**

| Number | Subject | Options | | |
| --- | --- | --- | --- | --- |
|  |  | True | False | Don’t know |
| 1 | Proper exercise can alleviate mental and psychological problems such as anxiety and depression. |  |  |  |
| 2 | The main reason for most mental and psychological problems lies in heredity. |  |  |  |
| 3 | Children are too stressed and lack exercise, which is not conducive to brain development. |  |  |  |
| 4 | To cultivate children's self-confidence, we should always praise children for their intelligence. |  |  |  |
| 5 | Strengthening social activities in the elderly can help slow down the decline of brain function. |  |  |  |
| 6 | Anxiety and other emotions are harmful. |  |  |  |
| 7 | Actively facing the things or environment that cause anxiety will help to gradually alleviate the anxiety problem. |  |  |  |
| 8 | The earlier the treatment of mental illness, the better. |  |  |  |
| 9 | Mental and psychological diseases can be alleviated and even recovered after effective treatment. |  |  |  |
| 10 | Looking at photos or videos of the accident or disaster scene may cause psychological trauma. |  |  |  |
| 11 | Compared with sudden trauma, continuous stress has little impact on mental and psychological health. |  |  |  |
| 12 | People who suffer from insomnia at night should sleep more during the day. |  |  |  |
| 13 | Drinking a small amount of alcohol before going to bed can help improve the quality of sleep. |  |  |  |
| 14 | Having a habit of cleanliness is an obsessive-compulsive disorder. |  |  |  |
| 15 | A bad mood may cause physical diseases. |  |  |  |
| 16 | Hypertension, coronary heart disease, and gastric ulcer are psychosomatic diseases. |  |  |  |
| 17 | Using the online psychological questionnaire, you can diagnose whether you have mental and psychological diseases. |  |  |  |
| 18 | It is easy to see whether a person has mental and psychological diseases. |  |  |  |
| 19 | The medical examination is normal, but he always suspects that he is ill, which may be a mental and psychological disease. |  |  |  |
| 20 | After taking medicine to improve mental and psychological diseases, you can reduce the amount of medicine while observing. |  |  |  |

**Part 2: Self-Assessment Questions**

| Number | Subject | Options | | | |
| --- | --- | --- | --- | --- | --- |
|  |  | Always  (Very agree) | Often  (Quite agree) | Sometimes  (Quite opposed) | Never  (Very opposed) |
| 1 | I am confident that I can overcome most of the difficulties in life. |  |  |  |  |
| 2 | I can deal with things rationally and keep calm. |  |  |  |  |
| 3 | I always keep a positive attitude toward life. |  |  |  |  |
| 4 | I know how to acquire mental health knowledge. |  |  |  |  |
| 5 | I know how to seek professional psychological help. |  |  |  |  |
| 6 | Mental health has a great impact on a person's physical health. |  |  |  |  |
| 7 | For a person, mental health is very important. |  |  |  |  |
| 8 | Everyone should learn some knowledge about mental health. |  |  |  |  |

**Part 3: Case Questions**

Here are two examples, which often happen around us. Please read carefully and understand the situation of the protagonist in the example, and answer the relevant questions according to your thoughts.

(1) Xiao Ming, male, 22 years old, a college student. He has been feeling sad in recent weeks. He thinks that he can't do anything well. He has failed to live, and can't see hope in life. He often suffers from insomnia at night, has no appetite for meals, and always feels tired and weak. He can't concentrate when studying and has no interest in his favorite football.

1. Do you think Xiao Ming's problem is a mental illness?

A. Yes B. Maybe C. No D. Don’t know

2. What do you think is the most likely problem for Xiao Ming? (Choose the most likely one)

A. Excessive stress B. Depression C. Schizophrenia D. Social anxiety

E. Stress disorder F. Obsessive-compulsive disorder

3. If you were Xiao Ming, what would you do?

| Questions | Options | | |
| --- | --- | --- | --- |
|  | Agree | Uncertain | Disagree |
| Don't tell others |  |  |  |
| Willing to talk to a psychological consultant |  |  |  |
| Willing to see a psychiatrist |  |  |  |

4. If Xiao Ming is diagnosed with a mental illness, do you agree with the following views?

| Questions | Options | | |
| --- | --- | --- | --- |
|  | Agree | Uncertain | Disagree |
| It's better to have less contact with him |  |  |  |
| It may be dangerous to be with him |  |  |  |
| He needs to be cared for more |  |  |  |

(2) Xiao Yu, female, 27 years old, a company employee. In the past year, when she participated in meetings, dinners with friends, and other activities, she became more and more afraid of talking to others. Whenever she spoke, she would become very nervous, her face would turn red, her hands would tremble, her heart would beat rapidly, and she could not even speak. But at home, she can express herself freely. Therefore, she dared not to meet strangers and tried her best to avoid all kinds of group activities.

1. Do you think Xiao Yu's problem is a mental illness?

A. Yes B. Maybe C. No D. Don’t know

2. What do you think is the most likely problem for Xiao Yu? (Choose the most likely one)

A. Excessive stress B. Depression C. Schizophrenia D. Social anxiety

E. Stress disorder F. Obsessive-compulsive disorder

3. If you were Xiao Yu, what would you do?

| Questions | Options | | |
| --- | --- | --- | --- |
|  | Agree | Uncertain | Disagree |
| Don't tell others |  |  |  |
| Willing to talk to a psychological consultant |  |  |  |
| Willing to see a psychiatrist |  |  |  |

4. If Xiao Yu is diagnosed with a mental illness, do you agree with the following views?

| Questions | Options | | |
| --- | --- | --- | --- |
|  | Agree | Uncertain | Disagree |
| It's better to have less contact with her |  |  |  |
| It may be dangerous to be with her |  |  |  |
| She needs to be cared for more |  |  |  |

**Scale Scoring and Judging Criteria**

The first part is the judgment questions: 20 questions in total, 5 points for each question. The total score range is 0-100 points. Among them, the correct answer to questions 1, 3, 5, 7, 8, 9, 10, 15, 16, and 19 should be "True"; the correct answer to questions 2, 4, 6, 11, 12, l3, 14, l7, 18 and 20 should be "False". Each judgment question is 5 points for the correct answer, 0 points for the wrong answer, or a *don't know* answer. The total score of the 20 questions is the direct sum of the scores.

The second part is the self-assessment questions: 8 questions in total, all with 1-4 scores. The total score range is 8-32 points. Option A *always/very agree* = 4 points, B *often/quite agree* = 3 points, C *sometimes/quite oppose* = 2 points, and D *never/very oppose* = 1 point. The scores of the 8 questions are directly summed to give the total score of the self-assessment questions.

The third part is the case questions: the total score range is 0-40 points. There are 2 groups of case questions, each group contains 4 questions, and each question is scored as follows. Question 1, option A *yes* = 6 points, B *maybe* = 2 points, C *no* = 0 points, and D *don’t know* = 0 points. Question 2, the correct option = 2 points, and the remaining options = 0 points. The correct option in case 1 is B *depression*, and the correct option in case 2 is D *social anxiety*. Question 3 contains three small questions, and the scores are as follows: "Don't tell others": *agree* = 0 points, *uncertain* = 1 point, *disagree* = 2 points; "Willing to talk to a psychological consultant": *agree* = 2 points, *uncertain* = 1 point, *disagree* = 0 points; "Willing to see a psychiatrist": *agree* = 2 points, *uncertain* = 1 point, *disagree* = 0 points. Question 4 also contains three small questions, and the scores are as follows: "It is better to have less contact with him/her": *agree* = 0 points, *uncertain* = 1 point, *disagree* = 2 points; "It may be dangerous to be with him/her": *agree* = 0 points, *uncertain* = 1 point, *disagree* = 2 points; "He/she needs to be cared for more": *agree* = 2 points, *uncertain* = 1 point, *disagree* = 0 points. The scores of all questions of the two cases in the third part are directly summed up, which is the total score of the case questions.

The mental health literacy of the same respondent, which is considered to be qualified, meets the following three conditions at the same time: (a) The total score of judgment questions is ≥ 80 points; (b) The total score of self-assessment questions is ≥ 24 points; (c) The total score of case questions is ≥ 28 points.

**Appendix S2**

**Table B1**

*Model Predicting Insomnia from Anxiety, Moderated by Older Adults’ and Caregivers’ Mental Health Knowledge, Attitudes and Capacity Respectively*

|  | Young Caregiver  (18-44 years old)  (*n* = 565) | | | Middle-aged Caregiver  (45-64 years old)  (*n* = 367) | | | Older Caregiver  (over 65 years old)  (*n* = 101) | | |
| --- | --- | --- | --- | --- | --- | --- | --- | --- | --- |
| Effect | *β* | *SE* | *P* | *β* | *SE* | *P* | *β* | *SE* | *P* |
| **Actor effect** |  |  |  |  |  |  |  |  |  |
| Older adult | **0.772** | **0.074** | **< .001** | **0.780** | **0.076** | **< .001** | **1.038** | **0.163** | **< .001** |
| Caregiver | **0.589** | **0.053** | **< .001** | **0.722** | **0.068** | **< .001** | **1.049** | **0.266** | **< .001** |
| **Partner effect** | |  |  |  |  |  |  |  |  |
| Older adult | -0.016 | 0.072 | .824 | -0.093 | 0.081 | .251 | -0.143 | 0.259 | .582 |
| Caregiver | 0.096 | 0.052 | .066 | 0.062 | 0.061 | .308 | -0.098 | 0.165 | .551 |
| **Older adult’s Anxiety by Older adult’s MHK** | | | | | | | | | |
| Older adult | 1.020 | 0.541 | .059 | 0.808 | 0.514 | .116 | 0.339 | 0.755 | .654 |
| Caregiver | 0.433 | 0.392 | .270 | -0.393 | 0.423 | .353 | 0.214 | 0.772 | .782 |
| **Older adult’s Anxiety by Caregiver’s MHK** | | | | | | | | | |
| Older adult | -0.148 | 0.193 | .443 | -0.402 | 0.488 | .410 | -^1^ | -^1^ | -^1^ |
| Caregiver | -0.201 | 0.140 | .153 | -0.037 | 0.395 | .926 | -^1^ | -^1^ | -^1^ |
| **Caregiver’s Anxiety by Older adult’s MHK** | | | | | | | | | |
| Older adult | -0.100 | 0.252 | .691 | 0.213 | 0.411 | .604 | -^1^ | -^1^ | -^1^ |
| Caregiver | **0.428** | **0.182** | **.019** | 0.122 | 0.340 | .720 | -^1^ | -^1^ | -^1^ |
| **Caregiver’s Anxiety by Caregiver’s MHK** | | | | | | | | | |
| Older adult | 0.032 | 0.244 | .896 | -0.148 | 0.536 | .782 | -2.569 | 3.772 | .497 |
| Caregiver | -0.110 | 0.177 | .535 | 0.264 | 0.436 | .545 | -3.013 | 3.898 | .441 |
| **Actor effect** |  |  |  |  |  |  |  |  |  |
| Older adult | 0.458 | 0.311 | .140 | **1.009** | **0.255** | **< .001** | 9.543 | 13.553 | .482 |
| Caregiver | **1.186** | **0.217** | **< .001** | **1.904** | **0.367** | **< .001** | -0.727 | 9.672 | .940 |
| **Partner effect** | |  |  |  |  |  |  |  |  |
| Older adult | -0.305 | 0.299 | .309 | **-0.914** | **0.457** | **.046** | -5.541 | 9.929 | .578 |
| Caregiver | 0.032 | 0.225 | .888 | -0.180 | 0.202 | .373 | 2.975 | 13.214 | .822 |
| **Older adult’s Anxiety by Older adult’s MHA** | | | | | | | | | |
| Older adult | 0.104 | 0.203 | .608 | 0.102 | 0.166 | .538 | -10.257 | 14.399 | .477 |
| Caregiver | -0.147 | 0.146 | .313 | 0.084 | 0.131 | .519 | -4.687 | 14.071 | .739 |
| **Older adult’s Anxiety by Caregiver’s MHA** | | | | | | | | | |
| Older adult | 0.220 | 0.268 | .412 | -0.307 | 0.262 | .243 | 1.786 | 1.469 | .226 |
| Caregiver | 0.187 | 0.194 | .335 | 0.161 | 0.208 | .440 | 1.681 | 1.488 | .260 |
| **Caregiver’s Anxiety by Older adult’s MHA** | | | | | | | | | |
| Older adult | 0.084 | 0.196 | .669 | 0.616 | 0.319 | .054 | 5.235 | 9.178 | .569 |
| Caregiver | -0.091 | 0.142 | .520 | **-0.731** | **0.254** | **.004** | 3.197 | 8.959 | .722 |
| **Caregiver’s Anxiety by Caregiver’s MHA** | | | | | | | | | |
| Older adult | 0.234 | 0.248 | .346 | 0.282 | 0.366 | .441 | -0.223 | 1.188 | .851 |
| Caregiver | **-0.558** | **0.180** | **.002** | -0.515 | 0.290 | .076 | -1.497 | 1.149 | .194 |
| **Actor effect** |  |  |  |  |  |  |  |  |  |
| Older adult | **0.762** | **0.164** | **< .001** | **0.935** | **0.131** | **< .001** | 1.289 | 0.713 | .073 |
| Caregiver | **0.664** | **0.103** | **< .001** | **0.601** | **0.121** | **< .001** | **2.105** | **0.574** | **< .001** |
| **Partner effect** | |  |  |  |  |  |  |  |  |
| Older adult | -0.054 | 0.142 | .704 | **-0.315** | **0.154** | **.041** | -0.502 | 0.585 | .392 |
| Caregiver | 0.149 | 0.118 | .207 | **0.234** | **0.103** | **.023** | 0.087 | 0.704 | .902 |
| **Older adult’s Anxiety by Older adult’s MHC** | | | | | | | | | |
| Older adult | -0.167 | 0.136 | .218 | -0.078 | 0.157 | .622 | 0.041 | 0.515 | .936 |
| Caregiver | -0.055 | 0.098 | .575 | 0.092 | 0.125 | .461 | -0.357 | 0.504 | .480 |
| **Older adult’s Anxiety by Caregiver’s MHC** | | | | | | | | | |
| Older adult | 0.127 | 0.171 | .458 | -0.187 | 0.161 | .246 | -0.262 | 0.777 | .736 |
| Caregiver | -0.041 | 0.123 | .737 | **-0.367** | **0.128** | **.004** | 0.240 | 0.761 | .753 |
| **Caregiver’s Anxiety by Older adult’s MHC** | | | | | | | | | |
| Older adult | 0.162 | 0.139 | .243 | 0.229 | 0.168 | .173 | 0.342 | 1.248 | .784 |
| Caregiver | 0.192 | 0.101 | .058 | 0.131 | 0.132 | .319 | 0.312 | 1.213 | .798 |
| **Caregiver’s Anxiety by Caregiver’s MHC** | | | | | | | | | |
| Older adult | -0.051 | 0.160 | .747 | 0.225 | 0.162 | .165 | 0.163 | 1.316 | .901 |
| Caregiver | -0.196 | 0.116 | .090 | 0.082 | 0.128 | .524 | -1.632 | 1.278 | .203 |

*Note.* Models adjusted for older adult’s/caregiver’s age, sex, region, and the number of chronic diseases. MHK = Mental Health Knowledge; MHA = Mental Health Attitudes; MHC = Mental Health Capacity. Significant effects are in bold.

^1^ "-" indicates that the parameter is redundant and equal to 0.
